# Local Deployment of Open-Weight Language Models in Dermatology: Viewpoint on Privacy, Equity, and Practical Implementation

**DOI:** 10.2196/94764

**Published:** 2026-08-21

**Authors:** William J Nahm, Emily S Yin, Emily C Milam, Jason G Weed

**Affiliations:** 1 Dr. Phillip Frost Department of Dermatology and Cutaneous Surgery, University of Miami Miller School of Medicine, 1295 N.W. 14th Street, Miami, FL, 33136, United States; 2 The Ronald O. Perelman Department of Dermatology, New York University Grossman School of Medicine, New York, NY, United States

**Keywords:** large language models, model compression, artificial intelligence, AI, dermatology, Health Insurance Portability and Accountability Act, HIPAA, teledermatology, local deployment, open-weight models, clinical documentation, data privacy

## Abstract

Generative AI, particularly large language models (LLMs), is reshaping clinical workflows in dermatology. However, cloud-based commercial models pose persistent challenges to Health Insurance Portability and Accountability Act (HIPAA) compliance, especially in dermatology, where protected health information (PHI) extends beyond text to clinical photographs, dermoscopic images, and total-body photography that may capture identifiable anatomical features and document conditions carrying social stigma. Locally hosted, open-weight LLMs that are run within the institution’s own infrastructure offer dermatology practices a pathway to leverage AI capabilities while retaining full control of their data. This viewpoint synthesizes evidence on when locally hosted, open-weight LLMs should be preferred for dermatologic workflows, when cloud deployment may remain preferable, and how multimodal AI fits into a coherent local deployment strategy. We advance 4 arguments. First, model compression techniques (knowledge distillation, structured pruning, and low-bit quantization) together with mixture-of-experts architectures have lowered hardware thresholds enough that 7- to 33-billion-parameter models now run on consumer-grade workstations with modest neural processing units or graphics processing units. Second, dermatology is fundamentally a visual specialty, and a credible local deployment strategy must integrate LLMs with vision models, including convolutional neural networks, vision transformers, vision-language models, and dermatology-specific foundation models such as PanDerm and medical multimodal models such as MedGemma. Third, locally hosted, open-weight models confer specific advantages for dermatology, including complete institutional control of clinical images, freedom from vendor model deprecation that disrupts validated workflows, and the ability to audit and fine-tune models to address well-documented performance gaps in skin of color. Fourth, local deployment is not a panacea; cloud models remain preferable for some tasks, and local deployment introduces governance challenges (heterogeneity across practices, model drift, and quantization-induced accuracy loss) that require structured mitigation through validated reporting frameworks such as CONSORT-AI (Consolidated Standards of Reporting Trials), SPIRIT-AI (Standard Protocol Items: Recommendations for Interventional Trials), DECIDE-AI (Developmental and Exploratory Clinical Investigations of Decision support systems driven by AI), and TRIPOD+AI (Transparent Reporting of a Multivariable Prediction Model for Individual Prognosis or Diagnosis), as well as retrieval-augmented generation and federated learning approaches. We situate these arguments within the international regulatory landscape, including the European Union’s General Data Protection Regulation, the European Union AI Act, and Germany’s *Digitale Gesundheitsanwendungen* (DiGA) framework, in addition to HIPAA. We provide quantitative cost examples showing that current consumer hardware capable of running 14- to 33-billion-parameter models can be acquired for roughly the price of 1 to 2 years of enterprise cloud-AI subscriptions. We close by mapping a practical implementation pathway and identifying near-term research priorities. Locally hosted, open-weight LLMs that are deployed thoughtfully and within governance frameworks offer dermatology practices a credible route to harness generative AI while preserving regulatory compliance, equity across skin types, and the dermatologist-patient relationship.

## Introduction

Generative AI, particularly large language models (LLMs), has considerable potential to enhance health care delivery, from generating patient summaries to assisting with medical coding and optimizing practice workflows [1]. In dermatology, AI applications are expanding rapidly across clinical image analysis, teledermatology, dermatopathology, patient education, and clinical decision support [2,3]. The dermatology field has witnessed exponential growth in deep learning research since 2016, culminating in the US Food and Drug Administration’s landmark 2024 approval of DermaSensor, the first AI skin cancer detection device authorized for primary care use [4].

Despite this momentum, the cloud-based deployment of leading commercial models, including OpenAI’s GPT-5.2, Anthropic’s Claude Opus 4.6, xAI’s Grok, and Google’s Gemini 3 Pro, raises substantive concerns about data security and Health Insurance Portability and Accountability Act (HIPAA) compliance [5]. HIPAA mandates that protected health information (PHI), including personally identifiable information, remain confidential and within the control of health care entities or verified business associates [6]. While cloud-based commercial LLMs can technically achieve HIPAA compliance through Business Associate Agreements (BAAs) with vendors, such as those available through enterprise tiers of OpenAI, Microsoft Azure, and Amazon Web Services, this approach introduces dependency on external vendors’ data governance policies, limited institutional autonomy over AI infrastructure, and vulnerability to pricing and policy changes [5]. Frequent update cycles of proprietary models compound these concerns; when closed models are updated to optimize performance for general audiences, those changes can disrupt validated clinical workflows, and model versions are routinely deprecated within months, requiring health care organizations to revalidate clinical tools built upon them [5].

These concerns are particularly acute in dermatology, where they extend beyond textual records to include clinical photographs, dermoscopic images, and total-body photography that may capture identifiable anatomical features [7]. Dermatologic images of conditions such as sexually transmitted infections, autoimmune blistering diseases, or psychodermatologic manifestations carry heightened sensitivity due to social stigma, making the privacy of these data a paramount clinical and ethical concern [8]. The risks of poor data stewardship in AI-driven dermatology were starkly illustrated by a 2021 case in which a United Kingdom–based teledermatology platform disclosed patient photographs without obtaining appropriate authorization [9]. Locally hosted, open-weight LLMs, deployed within the institution’s own infrastructure rather than transmitted to vendor cloud end points, present a compelling alternative for dermatology practices seeking to integrate generative AI while maintaining the highest standards of data protection. We propose that locally hosted, open-weight LLMs, when integrated with complementary vision models and embedded in adequate governance, may be the preferred deployment model for many dermatologic workflows. We articulate where this preference holds, where cloud models remain better suited, and what implementation steps clinical practices can realistically take.

The remainder of this viewpoint is organized as follows. We first describe our approach to the literature. We then consider open-weight model characteristics and the hardware thresholds at which locally hosted deployment becomes practical, followed by a discussion of multimodal AI relevant to dermatology. Subsequent sections address model compression and its dermatologic implications, practical local deployment infrastructure, and scenarios in which cloud-based deployment may remain preferable. We then examine regulatory frameworks beyond the HIPAA, review performance evidence and benchmarking, and discuss bias, equity, and the specific role of open-weight models in dermatology. We close with governance, validation, and reporting standards, reliability and data quality considerations, risks of overreliance and mitigation strategies, and future directions.

## Methodological Approach to the Literature Review

We searched the PubMed, Embase, and arXiv databases between January 2020 and January 2026 using the terms “large language model,” “open-weight,” “local deployment,” “on-premises,” “vision-language model,” “foundation model,” “federated learning,” “dermatology,” “skin of color,” “HIPAA,” and “GDPR,” alone and in combination. Peer-reviewed primary research and consensus reports were prioritized. We also drew on official model documentation, hardware vendor technical disclosures, and reporting framework publications (CONSORT-AI [Consolidated Standards of Reporting Trials], SPIRIT-AI [Standard Protocol Items: Recommendations for Interventional Trials], DECIDE-AI [Developmental and Exploratory Clinical Investigations of Decision Support Systems driven by AI], and TRIPOD+AI [Transparent Reporting of a Multivariable Prediction Model for Individual Prognosis or Diagnosis]) to characterize evolving infrastructure and governance. Where non–peer-reviewed technical disclosures were used, they were cross-checked against peer-reviewed analyses; web pages were retained only where no peer-reviewed equivalent existed.

## Open-Weight Language Model Architectures and Hardware Requirements

Local deployment of AI solutions predominantly relies on open-weight LLMs, which provide trained model weights for inference while typically withholding full training methodology details [10]. The term “open-weight” is preferred here over “open-source,” as most popular models, including the Llama and Mistral families, do not meet the full criteria of open-source software, which would require disclosure of training code, data, and architecture [10]. A model being closed does not inherently preclude local deployment; closed models are typically unavailable for on-premises use because vendors do not distribute their weights for local inference. Notably, the landscape is shifting: in 2025, OpenAI released its first open-weight models for self-hosting, signaling that even developers of traditionally closed models now recognize the value of local deployment.

The advancement of LLMs has been driven by the continuous expansion of training datasets and parameter counts. For context, OpenAI’s GPT-3 comprised 175 billion parameters; competing open-weight LLMs now exceed 600 billion parameters (eg, DeepSeek V3.2). The latest generation of open-weight models increasingly use mixture-of-experts (MoE) architectures that activate only a subset of parameters per query. Alibaba’s Qwen 3 contains 235 billion total parameters but activates only 22 billion per token, reducing inference costs substantially compared with dense models of equivalent capability. Meta’s Llama 4 introduced natively multimodal MoE models with 17 billion active parameters across up to 128 experts, fitting on a single graphics processing unit (GPU) with 4-bit integer (INT4) quantization, and Mistral AI’s Mistral Large 3, released in December 2025 under the Apache 2.0 license, offers 41 billion active parameters within a 675-billion-parameter total MoE architecture. These developments are important precisely because they reduce the hardware threshold for clinically relevant inference.

Practical hardware thresholds, however, are not adequately captured by parameter counts alone. Table 1 summarizes representative open-weight model classes against memory footprint after INT4 quantization (a process to make an AI model smaller and faster by storing some of its numbers using INT4 instead of larger formats) and current hardware capable of running them at clinically usable speeds. A representative dermatology workstation, an Apple Mac Studio with M5 Max (64 GB unified memory, approximately US $3500 in early 2026) or a desktop with a single 24 GB consumer GPU (eg, NVIDIA RTX 5090, approximately US $2000), can currently run dense 14- to 33-billion-parameter models or MoE models with comparable active-parameter counts at interactive latencies. By contrast, enterprise cloud-AI subscriptions with HIPAA-eligible end points typically run US $30 to US $60 per user per month for clinical-grade tiers, yielding 3-year costs of US $1000 to US $2000 per clinician. For a 4-clinician dermatology practice, 2 years of cloud-AI subscriptions therefore approximate the one-time cost of a workstation capable of supporting the entire practice. Although a formal cost-effectiveness analysis is beyond the scope of this viewpoint, this comparison illustrates that the affordability argument is no longer hypothetical.

**Table 1.** Representative open-weight model classes and indicative hardware requirements after 4-bit integer (INT4) quantization^a^.

Model class (examples)	Active parameters (approximately)	Memory after INT4 (approximately)	Representative hardware	Suitable dermatology tasks
Llama 3 8B and Qwen 3 8B [11,12]	7‐8 billion	5 GB	Laptop with 16 GB RAM and modern NPU^b^ [13]	Note structuring, basic patient education, and deidentification [3,14,15]
Llama 4 Scout, Qwen 3 14B and Mistral Small 3 [12,16,17]	14‐17 billion	10‐12 GB	Workstation with single 24 GB GPU^c^ or M5 Pro/Max with 32‐64 GB [18,19]	Drafting clinical letters, teledermatology triage, and RAG^d^ over local references [20-22]
Qwen 3 32B and DeepSeek V3.2 (MoE^e^) [12,23]	22‐37 billion (active)	20‐30 GB	Workstation with 48 GB GPU or M5 Max/Ultra with 64‐128 GB [18,19]	Differential diagnosis drafting (with HITL^f^) and dermatopathology summaries [24,25]
Llama 4 Maverick and Mistral Large 3 (MoE) [16,26]	>41 billion (active)	40‐60 GB	Server with 1‐2 enterprise GPUs (eg, H100 or H200) or Blackwell Ultra [27]	Group-practice or department-wide deployment and multitenant inference [5,28,29]

a Memory estimates assume INT4 quantization and standard context windows; vision and embedding models add to the budget. Hardware configurations are from early 2026.

b NPU: neural processing unit.

c GPU: graphics processing unit.

d RAG: retrieval-augmented generation.

e MoE: mixture-of-experts.

f HITL: human-in-the-loop.

## Multimodal AI Systems for Dermatologic Applications

Dermatology is fundamentally a visual specialty, and the AI tools most relevant to dermatologic care span LLMs, image-only convolutional neural networks (CNNs), vision transformers, vision-language models (VLMs), and emerging dermatology-specific foundation models. Mehta et al [24] trace a clear evolution in dermatopathology from first-generation CNNs to hybrid CNN-transformer systems and large-scale foundation models such as Virchow and PanDerm, with several systems (eg, DermAI and PathAssist Derm) now in real-world deployment.

Yan et al [25] recently described PanDerm, a multimodal vision foundation model pretrained on more than 2 million skin disease images from 11 institutions across 4 imaging modalities. Across 28 benchmarks spanning skin cancer screening, differential diagnosis, lesion segmentation, and longitudinal monitoring, PanDerm achieved state-of-the-art performance, often outperforming existing models with as little as 10% of labeled data, and improved nondermatologist health care professionals’ differential diagnosis by 16.5% across 128 skin conditions on clinical photographs [25]. Although PanDerm is at the research stage, its architecture, model weights, and broader trajectory are directly relevant to local deployment, as a dermatology-specific foundation model that can be downloaded and fine-tuned on institution-controlled data is precisely the type of tool local deployment is designed to support. Medical multimodal LLMs such as Google’s MedGemma offer a complementary class of models combining vision and language capabilities under permissive licenses suitable for local deployment.

Two implications follow. First, hardware planning for local deployment must account not only for LLM memory footprints but also for vision-model inference budgets; a workstation provisioned to run a 14-billion-parameter LLM may need additional GPU memory to host a CNN ensemble or a VLM concurrently. Second, the regulatory and governance considerations developed below for LLMs largely apply, with appropriate adaptation, to local vision-model deployment as well. We therefore use “locally hosted models” inclusively in subsequent sections, while continuing to emphasize LLM-specific considerations where they differ.

## Model Compression for Dermatologic Applications

Model compression techniques have emerged as a solution to these computational and financial barriers by transforming resource-intensive general-purpose models into compact variants deployable on constrained hardware [30,31]. These techniques enable the distillation of large models into smaller variants optimized for specific clinical use cases, trading broad generalization for targeted performance in defined tasks [30]. Key approaches include knowledge distillation, pruning, and quantization [30,31]. Knowledge distillation transfers knowledge from a large, complex “teacher” model to a smaller, more efficient “student” counterpart, reducing computational requirements while preserving task-relevant performance [31]. Pruning eliminates nonessential parameters within neural networks, reducing storage requirements and inference costs [30]. Quantization compresses models by decreasing the bit precision of model weights, with INT4 quantization capable of reducing a 140 GB model to approximately 35 GB [30]. Hardware-accelerated quantization formats, such as NVIDIA 4-bit floating-point (NVFP4), introduced with the Blackwell Ultra GPU architecture in 2025, further optimize low-precision inference by approaching 8-bit floating-point accuracy at a substantially reduced memory footprint.

Quantization carries known costs that warrant explicit acknowledgment. Aggressive low-bit quantization can degrade performance, particularly on tasks requiring nuanced reasoning, such as differential diagnosis support for complex inflammatory dermatoses or interpretation of subtle dermoscopic features. The multidimensional safety evaluation of LLM compression by Xu et al [30] demonstrates that perplexity alone does not capture compression-induced safety degradations relevant to medical use, including increased rates of biased or harmful outputs. Newer quantization techniques (eg, Generative Pre-trained Transformer Quantization, Activation-aware Weight Quantization, and NVFP4) have narrowed but have not closed this gap. Practices should therefore (1) prefer the highest-precision variant their hardware supports for clinical tasks, (2) validate the specific quantized variant they deploy on dermatology-relevant prompts before clinical use, and (3) revalidate when changing quantization formats or hardware. For text-based clinical documentation tasks, such as generating dermatologic consultation notes or summarizing pathology reports, knowledge distillation typically preserves high accuracy while significantly reducing parameter counts, and accuracy losses from INT4 quantization are usually small. For diagnostic reasoning tasks, the performance gap between compressed and full-scale models may widen, and organizations should not assume that compression yields equivalent performance across all use cases without empirical validation [28,30].

## Infrastructure Requirements for On-Premises Model Deployment

A growing ecosystem of desktop applications, including LM Studio and Ollama, facilitates local deployment of small- and large-parameter open-weight LLMs on personal computers [32]. These applications provide intuitive graphical user interfaces for implementing and evaluating various LLMs on local hardware, eliminating dependence on external cloud computing. Neural processing units (NPUs) are specialized microprocessors designed to accelerate AI workloads, enabling efficient local inference without incurring cloud service costs [33]. Models in the Mistral, Llama, and Qwen families with 7 to 33 billion parameters now run efficiently on personal or small-office computers with 16 to 64 GB of memory, including Apple’s M5-series Mac models with embedded neural accelerators. The Apple M5 chip embeds neural accelerators directly within each GPU core, delivering meaningful improvements in time-to-first-token for dense 14-billion-parameter models on a laptop. The NVIDIA Jetson platform delivers cost-effective AI processing in compact, energy-efficient form factors for edge deployments.

At the data center and enterprise scale, NVIDIA’s Blackwell Ultra GB300 GPU offers 288 GB of HBM3e memory and substantial NVFP4 throughput, enabling hosting of 300-billion-plus parameter models without memory offloading. AMD’s Instinct MI350 series provides a competitive alternative for institutions seeking vendor diversity. Latency depends on 3 factors: model size, hardware match, and network conditions. For small-parameter models running on adequately provisioned local hardware, local latency is competitive with, and often better than, cloud alternatives because no network round-trip is required. For very large dense models on consumer hardware, cloud inference on specialized accelerators is typically faster. We therefore recommend that practices select a model size that matches their hardware, as a properly matched local deployment offers both privacy and competitive latency, while a mismatched one offers neither.

## Dermatology-Specific Use Cases and Clinical Workflows

**Figure 1.**
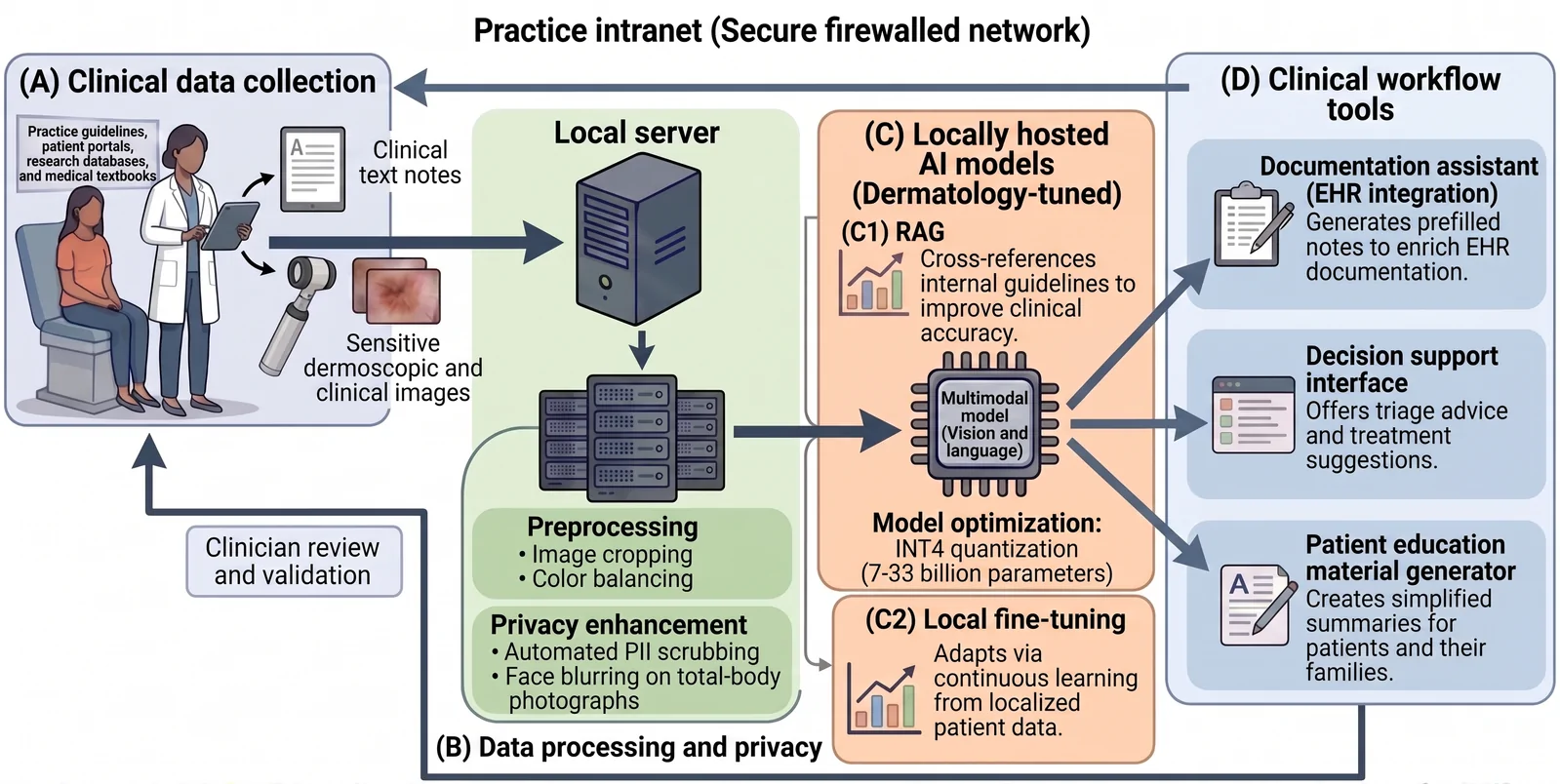
Operationalizing locally hosted, dermatology-tuned AI models (LLM plus vision) within a practice’s secure, firewalled intranet. (A) Clinical data collection: patient encounters and reference sources (practice guidelines, patient portals, research databases, and medical textbooks) generate clinical text notes and sensitive dermoscopic and clinical images that remain on local infrastructure. (B) Data processing and privacy: a local server preprocesses data (image cropping and color balancing) and applies privacy safeguards (automated personally identifiable information [PII] scrubbing and face blurring on total-body photographs). (C) Locally hosted AI models: a single multimodal (vision and language) model, quantized to 4-bit integer (INT4) at 7 to 33 billion parameters, draws on (C1) retrieval-augmented generation (RAG) against internal guidelines and (C2) local fine-tuning on localized patient data. (D) Clinical workflow tools: outputs power a documentation assistant for electronic health record (EHR) integration, a decision support interface offering triage and treatment suggestions, and a patient education material generator. A feedback loop returns model outputs to the clinician in panel A for human-in-the-loop review and validation before they are finalized or acted upon.

Local deployment creates powerful synergies with complementary technologies in dermatologic settings. A dermatology office can use specialized speech-to-text models to transcribe physicians’ dictation during skin examinations, including detailed descriptions of lesion morphology, distribution patterns, and dermoscopic features. These transcriptions can then be processed by local LLMs that transform raw conversational text into structured clinical notes conforming to dermatologic documentation standards. By deploying these systems entirely on local hardware, practices eliminate cloud-based processing of sensitive clinical descriptions and images, avoid service interruptions from third-party cloud downtime, and address patient concerns about external access to descriptions of their skin conditions. Source recordings could be retained and encrypted in local storage for a defined verification window. Where downstream queries of the documentation are needed, retrieval-augmented generation (RAG) over the institution’s own structured note repository (and, where appropriate, curated dermatologic references) should be preferred over reliance on the LLM’s parametric memory, as RAG materially reduces hallucination rates and provides citable provenance [34]. Figure 1 illustrates this end-to-end local workflow, from data capture through documentation, retrieval, and human review.

Beyond clinical documentation, locally deployed LLMs offer dermatology-specific applications across multiple domains. In teledermatology, local models can assist with triaging store-and-forward consultations by generating preliminary assessments from submitted images and clinical histories, reducing dermatologist review time while keeping all patient data within the institution [3,20]. For dermatopathology, small-parameter models can assist in structuring pathology reports, cross-referencing diagnostic criteria, and generating educational summaries for referring clinicians [24]. In the management of chronic inflammatory conditions such as psoriasis and atopic dermatitis, local AI can support treatment documentation, track biologic therapy responses, and generate patient-facing educational materials tailored to individual treatment plans [21]. Empirical evidence specifically supports LLM use for dermatologic diagnostic assistance, although with important caveats. In a comparative study using 100 histopathology-confirmed dermoscopic images from the International Skin Imaging Collaboration archive, Liu et al [35] reported that Claude 3 Opus and ChatGPT (GPT-4) achieved primary diagnostic accuracies of 56% and 48%, respectively, with the correct diagnosis appearing among the top 3 differentials in 76% and 78% of cases. Such performance is not adequate for autonomous diagnosis, but it indicates that current-generation LLMs (including locally deployable open-weight successors) can plausibly support shortlist generation, second-opinion prompts, and patient education when used with human-in-the-loop validation.

## Comparative Analysis of Locally Hosted and Cloud-Based Deployment Models

Local deployment is not a universal answer. We outline 4 scenarios in which cloud-based deployment, with appropriate BAAs, may be the better choice for a dermatology practice. First, when a workflow requires the highest available reasoning performance, frontier proprietary models running in vendor data centers continue to outperform locally deployable open-weight alternatives on the most demanding diagnostic reasoning tasks. Second, when a practice cannot reliably maintain on-premises infrastructure (no IT staff, frequent power interruptions, or aging hardware), cloud reliability may exceed what local deployment can offer. Third, when a workflow requires elastic burst capacity (eg, processing thousands of teledermatology cases during a public health surge), cloud resources scale more easily than fixed local hardware. Fourth, model drift, the gradual divergence of a deployed model’s performance from validation due to changes in patient population, documentation styles, or disease prevalence, is not solved by local deployment; if anything, isolated local deployments without centralized monitoring can experience drift unobserved for longer. Practices should weigh these considerations and may reasonably adopt hybrid architectures that route routine documentation locally and challenging diagnostic queries to a HIPAA-compliant cloud end point with explicit consent. Table 2 summarizes when each deployment model is preferable.

**Table 2.** When to prefer locally hosted versus cloud-based large language model deployment in dermatology.

Scenarios	Prefer locally hosted	Consider cloud-based (with BAA^a^)
Routine documentation and encounter summarization	Yes; sensitive content remains on premises [6]	Acceptable if BAA is in place and audited [6]
Stigmatized conditions (eg, sexual health and psychodermatology)	Strongly preferred [8]	Avoid unless clearly justified
Frontier diagnostic reasoning queries	Possible with ≥32 billion parameters and MoE^b^ if hardware permits	Often preferable; cloud frontier models still lead
Burst capacity (mass triage and public health surge)	Limited by fixed local hardware	Cloud elasticity advantageous
Practice without on-site IT support	Risk of unmaintained models and drift	Vendor-managed cloud may be more reliable
Cross-border (GDPR^c^ and European Union AI Act) workflows	Helps with data localization obligations [34]	Requires careful data processing agreements [34]

a BAA: Business Associate Agreement.

b MoE: mixture-of-experts.

c GDPR: General Data Protection Regulation.

## International Regulatory Frameworks and Cross-Jurisdictional Considerations

Although HIPAA shapes US dermatology practice, the international regulatory landscape is broader and increasingly relevant for cross-border teledermatology, multisite research, and academic-industry collaboration. The European Union’s General Data Protection Regulation (GDPR) imposes principles of data minimization, purpose limitation, and subject rights (including the right of access and erasure) that frequently align well with local deployment architectures. Data that never leave the institution is more readily reconciled with these obligations than data shared with third-country processors [34]. The European Union AI Act (Regulation EU 2024/1689) imposes additional obligations on high-risk AI systems used in health care, including risk management, data governance, transparency, and human oversight requirements, and requires conformity assessment under the Medical Device Regulation when the AI system constitutes a medical device. Germany’s *Digitale Gesundheitsanwendungen* (DiGA) framework, which provides reimbursement for prescription-grade digital health applications, illustrates how local deployment architectures can intersect with national reimbursement pathways. For example, DiGA-listed applications must satisfy stringent data protection requirements that local processing helps to meet.

For dermatology practices that operate across borders or partner with European researchers, 3 points are practically salient. First, local deployment is helpful but not sufficient; GDPR and the European Union AI Act impose obligations (documentation, conformity assessment, and postmarket monitoring) regardless of where computation occurs. Second, regulatory frameworks increasingly converge on transparency, auditability, and human oversight, all of which are easier to achieve with open-weight models. Third, locally hosted models can also raise their own questions about user input parameters; data the user types into a locally hosted system may be retained for fine-tuning, audited internally, or inadvertently exposed through weak access controls. Local deployment shifts the locus of risk from external transmission to internal governance, and practices must design access controls, audit trails, and prompt-logging policies to match [36].

## Performance Evidence and Benchmarking

Recent research demonstrates that locally deployed models can achieve strong performance in health care–relevant tasks. The LLM-Anonymizer study showed that local Llama 3 models achieved accuracies and sensitivities exceeding 98% for medical document deidentification, with both the 8-billion- and 70-billion-parameter variants performing comparably [14]. These findings suggest that smaller models can match larger counterparts for well-defined tasks, enabling deployment on standard modern hardware without substantial infrastructure investment.

However, comprehensive performance benchmarking across dermatologic use cases remains limited. Existing data focus primarily on document deidentification, and performance metrics for tasks such as diagnostic assistance, clinical note generation, and dermatopathology report summarization are lacking. The field urgently needs comparative data quantifying precision, recall, and clinical efficiency gains for locally deployed models versus commercial alternatives in dermatology-specific scenarios. Drawing on adjacent specialties, Luo et al [22] demonstrated that a locally hosted 13-billion-parameter model with retrieval augmentation achieved performance comparable to larger models in ophthalmology, noting that models smaller than 13 billion parameters may lack proficiency for complex medical reasoning but remain viable for many clinical and research applications. Analysis of LLM use patterns in health care shows that studies using open-weight models frequently focus on mental health applications, conversational agents, and documentation support, suggesting these models currently offer their strongest value proposition for customizable, privacy-sensitive workflows rather than high-complexity diagnostic reasoning [10]. Independent benchmarking platforms such as the Chatbot Arena and Open LLM Leaderboard show that leading open-weight models now approach the performance of proprietary models on many reasoning tasks, a trajectory that bodes well for clinical applications.

## Algorithmic Bias, Health Equity, and the Role of Open-Weight Models in Dermatologic Practice

The biases embedded within training data demand critical examination, particularly in dermatology, where these biases have well-documented and quantitatively measurable clinical consequences. Daneshjou et al [37] evaluated dermatology AI on the Diverse Dermatology Images set and reported substantial accuracy drops for darker skin tones, with state-of-the-art deep learning models classifying skin lesions less accurately on Fitzpatrick types V and VI than on lighter skin types. A subsequent scoping review of skin cancer image datasets found pervasive underrepresentation of darker skin tones and other minoritized groups across publicly available datasets, with limited or absent metadata on Fitzpatrick type, ethnicity, or geographic origin in many datasets [38]. A more recent dataset review by Wen et al [39] confirmed that dataset shifts arising from demographic variations and inconsistent labeling continue to compromise clinical utility and propagate discriminatory biases when algorithms are implemented in mismatched populations. Algorithmic bias resulting from feature selection and model weighting decisions can amplify these training data biases, intensifying health care disparities when AI systems are deployed in dermatologic practice [40].

These factors strengthen the argument for open-weight models in dermatology in 3 concrete ways that go beyond general claims of “transparency.” First, accessibility of weights enables institutions to fine-tune models on locally curated, demographically representative datasets, addressing population-specific performance gaps that vendors of closed models may not prioritize. Second, weight access permits empirical bias auditing through controlled probing, including stratified accuracy testing across Fitzpatrick types, which is impossible with closed models accessed only through APIs. Third, federated learning approaches over open-weight models enable multi-institutional fine-tuning to broaden representation without centralizing sensitive imaging data. Haggenmüller et al [41] demonstrated this approach concretely for melanoma versus nevus classification across 6 German university hospitals, with federated training achieving performance comparable to centralized training while preserving institutional data sovereignty. The dermatology community has both the opportunity and the obligation to use these capabilities deliberately so that AI tools serve patients across all skin types equitably [10].

## Governance Frameworks, Validation Protocols, and Reporting Standards for Clinical AI

While local deployment presents compelling opportunities, health care organizations must carefully evaluate inherent trade-offs. Compressed models may exhibit degradation in downstream task performance and amplification of safety concerns related to information accuracy, bias, and the generation of harmful content [30]. These evaluation challenges compound medicolegal uncertainties, as physicians, including dermatologists, lack clear guidance on incorporating AI technologies without increasing liability exposure [42,43]. Established AI reporting and evaluation frameworks should anchor local deployments. CONSORT-AI and SPIRIT-AI provide standards for AI-related randomized trials and trial protocols, TRIPOD+AI standardizes reporting of AI prediction model studies, and DECIDE-AI provides reporting guidance for early clinical evaluation of AI-driven decision support systems [44,45]. Although none of these frameworks were written specifically for locally deployed open-weight LLMs, their items (data provenance, model versioning, performance reporting across subgroups, and human-AI interaction documentation) translate directly to local deployment and should be incorporated into dermatology practice protocols and academic publications.

The possibility of numerous dermatology practices independently deploying different small models creates governance challenges that benefit from concrete, feasible mechanisms. We propose a tiered governance approach. At the practice level, every locally deployed model should have a model card recording the weights identifier, quantization, hardware, deployment date, intended use, validation dataset, and contraindications, plus an audit log capturing prompts, outputs, clinician overrides, and adverse events. At the network level, regional dermatology associations or academic consortia could host opt-in registries that aggregate model cards and adverse event signals; participation requires only minimal infrastructure (a shared form, a data-use agreement, and quarterly reporting) and could leverage federated approaches to share statistical signals without sharing PHI. At the national level, frameworks such as the Coalition for Health AI’s blueprint for trustworthy AI implementation, originally designed for centrally deployed systems, can be adapted to provide guidance and benchmarking standards for distributed local deployments [46]. We acknowledge that smaller practices may lack data scientists; this is precisely why federated learning models with explicit, prescriptive data-processing guidelines are more realistic than expecting each practice to design its own monitoring program [41].

## Reliability and Data Quality

Most LLMs lack health care–specific design or training. Unlike established clinical resources such as UpToDate, which use peer-reviewed content and expert curation, LLMs typically train on vast, unvetted web data without distinguishing reliable from questionable sources [47]. High-quality training data are optimal, yet the field has not established standardized thresholds for data reliability, as requirements vary substantially based on training data complexity and the intended clinical use case [48].

LLMs frequently lack transparent citation trails, and generated references may fail to support content or prove entirely fictional, leaving dermatologists unable to verify AI-generated clinical information [42]. The probabilistic nature of text generation introduces further reliability concerns; identical queries can produce varying responses across attempts, with outputs influenced by query phrasing and context [42]. Such variability can generate interaction biases affecting both clinical performance and objectivity [40]. The “black box” nature of machine learning algorithms compounds trust deficits, with global research indicating that 63% of participants express unwillingness or ambivalence toward trusting AI in health care [49,50]. Implementing human-in-the-loop frameworks where dermatologists validate AI outputs is crucial for mitigating these limitations [15]. Explainable AI technologies offer promising solutions; notably, Chanda et al [51] demonstrated through eye-tracking that dermatologist-like explainable AI enhanced melanoma diagnostic accuracy, suggesting that advances in interpretability can directly benefit dermatologic practice.

## Automation Bias and Mitigation Strategies

Clinician overreliance on LLMs poses documented risks. In a multicenter randomized clinical vignette study, clinicians experienced an 11.3% decrease in diagnostic accuracy when shown systematically biased AI model predictions, demonstrating how uncritical acceptance of AI outputs can compromise clinical judgment [52]. In dermatology specifically, nonspecialists may increasingly use AI tools for diagnosis despite lacking expertise in comprehensive management, a concern particularly relevant given the Food and Drug Administration’s recent approval of AI devices for primary care skin cancer screening [53,54]. Such overreliance threatens to diminish diagnostic competence and compromise essential pattern-recognition skills central to dermatologic practice [55].

Addressing these risks requires multifaceted strategies. Greater investment in human-computer interaction research within dermatology is needed to understand how clinicians engage with AI recommendations and where decision-making is most vulnerable to automation bias [52]. Mandatory human-in-the-loop validation protocols should be integrated into all clinical AI deployments, and locally hosted LLMs are well-suited to enforce these workflows in practice (eg, by requiring clinician acknowledgment before downstream actions). Additionally, research examining the impact of AI tools on patient trust and willingness to disclose information, particularly regarding sensitive skin conditions, is essential for guiding technical design. Patient perceptions matter not only ethically but also operationally. If patients perceive AI tools as protecting their privacy, they may be more forthcoming about stigmatized conditions, ultimately supporting clinical accuracy.

## Future Directions and Conclusions

Computational requirements for locally hosted models are expected to decrease as hardware advances and model compression techniques mature, particularly with continued progress in NPU capabilities and the open-weight ecosystem. Concurrently, the open-weight ecosystem continues to mature, with organizations including Meta, Alibaba, Mistral AI, DeepSeek, and OpenAI contributing high-performance models under permissive licenses, ensuring that locally deployable alternatives keep pace with proprietary offerings.

For dermatology specifically, 4 near-term priorities follow from this analysis. First, dermatology-specific benchmarking suites are needed that test locally deployable models on documentation, triage, and visual reasoning tasks, with performance stratified by Fitzpatrick type and condition rarity. Second, structured cost-effectiveness analyses should compare local and cloud deployments under realistic dermatology practice assumptions, including IT staffing and incident response costs. Third, federated learning collaborations across academic dermatology centers should be expanded, building on emerging precedents in melanoma diagnostics, to develop fine-tuned open-weight models that better represent diverse skin types without centralizing PHI [41]. Fourth, dermatology professional societies should issue practical guidance on model cards, audit logs, and adverse event reporting that smaller practices can realistically adopt.

## Abbreviations

BAA: Business Associate Agreement
CNN: convolutional neural network
CONSORT: Consolidated Standards of Reporting Trials
DECIDE: Developmental and Exploratory Clinical Investigations of Decision Support Systems
DiGA: *Digitale Gesundheitsanwendungen*
GDPR: General Data Protection Regulation
GPU: graphics processing unit
HIPAA: Health Insurance Portability and Accountability Act
INT4: 4-bit integer
LLM: large language model
MoE: mixture-of-experts
NPU: neural processing unit
NVFP4: NVIDIA 4-bit floating-point
PHI: protected health information
RAG: retrieval-augmented generation
SPIRIT: Standard Protocol Items: Recommendations for Interventional Trials
TRIPOD: Transparent Reporting of a Multivariable Prediction Model for Individual Prognosis or Diagnosis
VLM: vision-language model

## References

[1] Whig P, Dutta PK, Yathiraju N, Bhattacharya P, Sharma S, Bhattacharya P, Liu H, Dutta PK, Rodrigues JJ, Sethi G (2024). Revolutionizing Healthcare 5.0: The Power of Generative AI.

[2] Omiye JA, Gui H, Daneshjou R, Cai ZR, Muralidharan V (2023). Principles, applications, and future of artificial intelligence in dermatology. Front Med (Lausanne).

[3] Nahm WJ, Sohail N, Burshtein J, Goldust M, Tsoukas M (2025). Artificial intelligence in dermatology: a comprehensive review of approved applications, clinical implementation, and future directions. Int J Dermatol.

[4] Venkatesh KP, Kadakia KT, Gilbert S (2024). Learnings from the first AI-enabled skin cancer device for primary care authorized by FDA. NPJ Digit Med.

[5] Dennstädt F, Hastings J, Putora PM, Schmerder M, Cihoric N (2025). Implementing large language models in healthcare while balancing control, collaboration, costs and security. NPJ Digit Med.

[6] Marks M, Haupt CE (2023). AI chatbots, health privacy, and challenges to HIPAA compliance. JAMA.

[7] Yadav N, Pandey S, Gupta A, Dudani P, Gupta S, Rangarajan K (2023). Data privacy in healthcare: in the era of artificial intelligence. Indian Dermatol Online J.

[8] Gordon ER, Trager MH, Kontos D (2024). Ethical considerations for artificial intelligence in dermatology: a scoping review. Br J Dermatol.

[9] Zbrzezny AM, Krzywicki T (2025). Artificial intelligence in dermatology: a review of methods, clinical applications, and perspectives. Appl Sci.

[10] Xu J, Ding Y, Bu Y (2025). Position: open and closed large language models in healthcare. arXiv.

[11] (2024). Introducing Meta Llama 3: the most capable openly available LLM to date. Meta.

[12] (2025). Qwen3: think deeper, act faster. Qwen Studio.

[13] (2025). Apple unleashes M5, the next big leap in AI performance for Apple silicon. Apple.

[14] Wiest IC, Leßmann ME, Wolf F (2025). Deidentifying medical documents with local, privacy-preserving large language models: the LLM-anonymizer. NEJM AI.

[15] Bakken S (2023). AI in health: keeping the human in the loop. J Am Med Inform Assoc.

[16] (2025). The Llama 4 herd: the beginning of a new era of natively multimodal AI innovation. Meta AI.

[17] (2025). Mistral Small 3. Mistral AI.

[18] (2026). Apple introduces MacBook Pro with all‑new M5 Pro and M5 Max, delivering breakthrough pro performance and next-level on-device AI. Apple Inc.

[19] GeForce RTX 5090 graphics cards. NVIDIA.

[20] Shapiro J, Lyakhovitsky A (2024). Revolutionizing teledermatology: exploring the integration of artificial intelligence, including Generative Pre-trained Transformer chatbots for artificial intelligence-driven anamnesis, diagnosis, and treatment plans. Clin Dermatol.

[21] Jairath N, Pahalyants V, Shah R, Weed J, Carucci JA, Criscito MC (2024). Artificial intelligence in dermatology: a systematic review of its applications in melanoma and keratinocyte carcinoma diagnosis. Dermatol Surg.

[22] Luo MJ, Pang J, Bi S (2024). Development and evaluation of a retrieval-augmented large language model framework for ophthalmology. JAMA Ophthalmol.

[23] (2025). DeepSeek-V3.2 release. DeepSeek API Docs.

[24] Mehta A, Motavaf M, Raza D (2025). Deep learning image processing models in dermatopathology. Diagnostics (Basel).

[25] Yan S, Yu Z, Primiero C (2025). A multimodal vision foundation model for clinical dermatology. Nat Med.

[26] (2025). Introducing Mistral 3. Mistral AI.

[27] NVIDIA H100 GPU. NVIDIA.

[28] Shah NH, Entwistle D, Pfeffer MA (2023). Creation and adoption of large language models in medicine. JAMA.

[29] Umeton R, Kwok A, Maurya R (2024). GPT-4 in a cancer center — institute-wide deployment challenges and lessons learned. NEJM AI.

[30] Xu Z, Gupta A, Li T, Bentham O, Srikumar V (2024). Findings of the Association for Computational Linguistics: EMNLP 2024.

[31] Yang C, Zhu Y, Lu W (2025). Survey on knowledge distillation for large language models: methods, evaluation, and application. ACM Trans Intell Syst Technol.

[32] (2025). Local LLM deployment: privacy-first AI complete guide. Digital Applied.

[33] Whig P, Dutta PK, Yathiraju N, Bhattacharya P, Batra I, Bhattacharya P, Liu H, Dutta PK, Rodrigues JJ, Sethi G (2025). Revolutionizing Healthcare 5.0: The Power of Generative AI.

[34] Knott M, Krebs M, Kerscher A (2026). Large language models in healthcare quality management: a European perspective on process automation and compliance. Front Digit Health.

[35] Liu X, Duan C, Kim MK (2024). Claude 3 Opus and ChatGPT with GPT-4 in dermoscopic image analysis for melanoma diagnosis: comparative performance analysis. JMIR Med Inform.

[36] Murdoch B (2021). Privacy and artificial intelligence: challenges for protecting health information in a new era. BMC Med Ethics.

[37] Daneshjou R, Vodrahalli K, Novoa RA (2022). Disparities in dermatology AI performance on a diverse, curated clinical image set. Sci Adv.

[38] Wen D, Khan SM, Ji Xu A (2022). Characteristics of publicly available skin cancer image datasets: a systematic review. Lancet Digit Health.

[39] Wen D, Soltan A, Trucco E, Matin RN (2024). From data to diagnosis: skin cancer image datasets for artificial intelligence. Clin Exp Dermatol.

[40] Hanna MG, Pantanowitz L, Jackson B (2025). Ethical and bias considerations in artificial intelligence/machine learning. Mod Pathol.

[41] Haggenmüller S, Schmitt M, Krieghoff-Henning E (2024). Federated learning for decentralized artificial intelligence in melanoma diagnostics. JAMA Dermatol.

[42] Mello MM, Guha N (2023). ChatGPT and physicians’ malpractice risk. JAMA Health Forum.

[43] Nelson CA, Pachauri S, Balk R (2021). Dermatologists’ perspectives on artificial intelligence and augmented intelligence - a cross-sectional survey. JAMA Dermatol.

[44] Shiferaw KB, Roloff M, Balaur I, Welter D, Waltemath D, Zeleke AA (2025). Guidelines and standard frameworks for artificial intelligence in medicine: a systematic review. JAMIA Open.

[45] Frutuoso J (2025). Building a safe and transparent workflow for large language model (LLM)-assisted clinical trials and prediction models: a technical report. Cureus.

[46] (2023). Blueprint for trustworthy AI implementation guidance and assurance for healthcare. https://assets.ctfassets.net/7s4afyr9pmov/4AXIWGIlcrjWDaW2ueTaRS/f98e5cb2528187635895cce6ba5ec309/Blueprint_for_Trustworthy_AI.pdf.

[47] Harrer S (2023). Attention is not all you need: the complicated case of ethically using large language models in healthcare and medicine. EBioMedicine.

[48] Al Hleewa SO, Al Mubarak M, Al Mubarak M, Hamdan A (2023). Technological Sustainability and Business Competitive Advantage.

[49] Shevtsova D, Ahmed A, Boot IW (2024). Trust in and acceptance of artificial intelligence applications in medicine: mixed methods study. JMIR Hum Factors.

[50] Gillespie N, Lockey S, Ward T, Macdade A, Hassed G (2025). Trust, attitudes and use of artificial intelligence: a global study 2025. https://kpmg.com/in/en/insights/2025/05/trust-attitudes-and-use-of-ai.html.

[51] Chanda T, Haggenmueller S, Bucher TC (2025). Dermatologist-like explainable AI enhances melanoma diagnosis accuracy: eye-tracking study. Nat Commun.

[52] Jabbour S, Fouhey D, Shepard S (2023). Measuring the impact of AI in the diagnosis of hospitalized patients: a randomized clinical vignette survey study. JAMA.

[53] Khera R, Simon MA, Ross JS (2023). Automation bias and assistive AI: risk of harm from AI-driven clinical decision support. JAMA.

[54] Daneshjou R, Smith MP, Sun MD, Rotemberg V, Zou J (2021). Lack of transparency and potential bias in artificial intelligence data sets and algorithms: a scoping review. JAMA Dermatol.

[55] Quinn TP, Senadeera M, Jacobs S, Coghlan S, Le V (2021). Trust and medical AI: the challenges we face and the expertise needed to overcome them. J Am Med Inform Assoc.

